# Climate change and health risks in Mukuru informal settlement in Nairobi, Kenya – knowledge, attitudes and practices among residents

**DOI:** 10.1186/s12889-023-15281-y

**Published:** 2023-02-25

**Authors:** Johanne Greibe Andersen, Per Kallestrup, Catherine Karekezi, Gerald Yonga, Christian Kraef

**Affiliations:** 1 Center for Global Health, Department of Public Health, Aarhus University, Aarhus, Denmark; 2 Danish Non-communicable Diseases Alliance, Copenhagen, Denmark; 3 Kenya Diabetes Management and Information Centre, Nairobi, Kenya; 4 Non-communicable Diseases Alliance of Kenya, Nairobi, Kenya; 5 School of Medicine, University of Nairobi, Nairobi, Kenya; 6 Heidelberg Institute of Global Health, University of Heidelberg, Heidelberg, Germany; 7 Department of Infectious Diseases, Rigshospitalet, University of Copenhagen, Copenhagen, Denmark

**Keywords:** Climate change, Health Knowledge, attitudes, practice, Informal settlements, Slums, Non-communicable diseases, Communicable diseases, Low- and middle-income countries

## Abstract

**Background:**

Residents of informal settlements in Sub-Sahara Africa (SSA) are vulnerable to the health impacts of climate change. Little is known about the knowledge, attitudes and practices (KAP) of inhabitants of informal settlements in SSA regarding climate change and its health impacts. The aim of this study was to investigate how inhabitants of an informal settlement in SSA experience climate change and its health impacts and assess related knowledge, attitudes and practices. The study was conducted in Mukuru informal settlement in Nairobi City County, Kenya.

**Methods:**

A cross-sectional study was conducted in September 2021 using a structured, semi-closed KAP questionnaire. Inclusion criteria were ≥ 18 years of age and living in one of the three main sections in Mukuru: Kwa Njenga, Kwa Reuben or Viwandani. By spinning a pen at the geographic centre of each section, a random direction was selected. Then, in every second household one individual was interviewed, creating a representative mix of ages and genders of the local community. To assess participant characteristics associated with climate change knowledge multivariable logistic regression was used. Thematic content analysis was performed for qualitative responses.

**Results:**

Out of 402 study participants, 76.4% (*n* = 307) had heard of climate change before the interview, 90.8% (*n* = 365) reported that climate change was affecting their community, and 92.6% (*n* = 372) were concerned with the health-related impact of climate change. Having lived in Mukuru for more than 10 years and living in a dwelling close to the riverside were factors significantly associated with having heard of climate change before (aOR 3.1, 95%CI 1.7 – 5.8 and aOR 2.6, 95%CI 1.1 – 6.1, respectively) and experiencing a climate change related impact on the community (aOR 10.7, 95%CI 4.0 – 28.4 and aOR 7.7; 95%CI 1.7 – 34.0, respectively). Chronic respiratory conditions, vector-borne diseases, including infectious diarrhoea, malnutrition and cardiovascular diseases were identified by respondents as climate related health risks.

**Conclusions:**

Most respondents were knowledgeable about climate change and were experiencing its (health-related) impact on their community. This study provides insights which may prove useful for policy makers, intervention planners and researchers to work on locally adapted mitigation and adaption strategies.

## Background

Climate change is one of the major global health challenges of our time [1, 2]. According to the World Health Organization (WHO) climate change is indirectly and directly affecting many determinants of health including amongst others air quality, food security, access to clean water and secure shelter [3, 4]. Between 2015 and 2030 climate change is expected to cause approximately 250.000 additional deaths per year globally from malaria, diarrhoea, malnutrition, and heat stress alone [5, 6]. Residents in informal settlements in Low- and Middle-Income Countries (LMICs), including in Sub-Saharan Africa (SSA), are disproportionally affected by climate change and related health risks [7, 8]. The residents of informal settlements are more vulnerable to the health effects of climate change due to poor housing, pre-existing health issues and lack of basic infrastructure, including health care [9]. Furthermore, this group is often neglected in research and policy making, leading to a lack of quantitative and qualitative data for those most vulnerable [10, 11]. It is estimated that 1 billion people live in slum areas worldwide [12].

A recent systematic review on the scope of the existing literature on climate change and health in urban informal settlements in LMICs found a limited body of evidence with very few original research articles from the WHO African Region. A recurring theme among the reviewed articles was the need to identify the most vulnerable groups as well as effective methods to understand the local needs and to involve citizens and community groups in climate adaptation strategies [7].

Evidence is scarce on knowledge, attitudes and practices regarding climate change and related health risks in informal settlements [9, 13]. In Vietnam a study found that 70.1% of study respondents that lived in slums had heard about climate change and its impact on human health, compared to 79.3% of respondents living in non-slum areas [14]. A study among vulnerable communities – defined as prone to climate change hazards like cyclone and flood – in Bangladesh found that 54.2% of respondents had some knowledge about climate change, and that people with higher educational level or who live near a school were more knowledgeable about climate change and its impact on health [15]. A Nepalese study performed in a district located in the central hilly region of the country reported 54.7% of respondents had perceived a change in climate, that poverty increased the likelihood of perceiving health risks while illiteracy decreased the likelihood of perceiving health risks [16]. A Tanzanian study found that rural communities have little knowledge on climate change and its impacts on e.g., malaria, and only one in four understood the Swahili term for climate change. However, there was a general understanding that the rain patterns have changed in the past 10 years [17]. A recent qualitative study on the perception of climate change related health risks among academia, policy makers, health care workers, and local volunteers and community leaders working in Mukuru informal settlement in Nairobi, Kenya identified important focal areas for further climate and environmental health adaptation strategies: food and general economic security, access to clean water, clean air, climate resilient urban planning and public infrastructure, and a specific focus on vulnerable groups such as children [18].

A thorough understanding of residents’ knowledge and perceptions regarding climate change and its related impacts in informal settlements is needed to support interventions aimed at mitigating health risks that are effective. This will support interventions and adaptation strategies that are culturally appropriate, and tailored to the local environmental context.

## Methods

### Study aim, design, and setting

The study was conducted in Mukuru informal settlement in Nairobi City County, Kenya. Mukuru fulfills the definition for an informal settlement set by UN-Habitat as:

“*One in which inhabitants suffer one or more of the following ‘household deprivations’: lack of access to improved water source, lack of access to improved sanitation facilities, lack of sufficient living area, lack of housing durability and lack of secure tenure*” [19].

In 2016, the three sections (Mukuru Kwa Njenga, Mukuru Kwa Reuben and Viwandani) in Mukuru informal settlement in Nairobi, Kenya were estimated to have in total 301,683 inhabitants, living in 100,562 households [20]. Conservative population projections of 6% annual growth estimate that in 2030 Mukuru will have 682,076 inhabitants [20]. Of residents in Mukuru, 92% are renters/tenants and the housing usually consists of 10 × 10 feet structures with walls and roof made of sheet metal, frequently with dirt floors [20].

This study aimed at investigating what the local residents in the Mukuru informal settlement (Mukuru Kwa Njenga, Mukuru Kwa Reuben and Viwandani) in Nairobi know, think and how they act (knowledge, attitudes and practices) in regard to climate change and climate change mediated health risks. The study design was cross-sectional using a semi-closed questionnaire. To ensure validity and reliability the data collection questionnaire was developed based on similar studies in other LMICs [21–24] and then modified to the Kenyan context with help from the Kenyan partners at the Non-Governmental Organization (NGO) the NCD Alliance of Kenya (NCDAK) and pilot tested twice by NCDAK. The pilot testing led to minor changes in the order of the questions to allow a natural flow in the interviews.

Sample size for the study was calculated using total population size of 301,683, a confidence level of 95%, a margin of error of 5% and a very conservative assumed response distribution of 50%. The target sample size was 384 participants. Based on the relative size of the three sections in Mukuru the target was to include 40% of participants from Mukuru Kwa Njenga, 35% from Mukuru Kwa Reuben, and 25% from Viwandani. Inclusion criteria for participants were being ≥18 years of age to be able to consent to study participation and living in the study area.

### Data sampling and collection

Data was collected in September 2021. Together with a local resident as guide the research assistants went in survey teams of two to three to the three main sections in Mukuru informal settlement: Mukuru Kwa Njenga, Mukuru Kwa Reuben and Viwandani. Survey teams went to the geographic centre of each section, and by spinning a pen selected a random direction. One household was then randomly selected, using a random number table, as the first to be included in the survey. Then every second household was surveyed. Only one individual from each household in the eligible age group was chosen by using the Kish method creating a representative mix of ages and genders of members of the local community. The questionnaires were conducted face-to-face inside or in front of the home of each respondent. Survey responses were collected and managed using the secure REDCap (Research Electronic Data Capture) data capture tool hosted at Aarhus University [25, 26]. Responses were noted manually by the research assistants.

The study participants were recruited from and lived in Mukuru Kwa Njenga 38.9% (*n* = 156), Mukuru Kwa Reuben 39.2% (*n* = 157) and Viwandani 21.9% (*n* = 88). The interviews were conducted in Swahili (81.1%), English (15.4%) or a mix of English and Swahili (3.5%). Research assistants, recruited with assistance from the NCDAK, performed the data collection and data entry.

### Data analysis

Quantitative data on characteristics of participants were presented using descriptive statistics.

To examine the associations between knowledge of climate change, experiencing that climate change affects the community and being concerned with the health-related impacts of climate change and patient characteristics bivariate analysis was performed using logistic regression. To control for confounding variables, multivariate logistic regression was used. We included those variables with a *p*-value ≤0.1 in the multivariable model. Odds ratios (ORs), corresponding 95% confidence intervals (CIs) and *p*-values were calculated for bivariate and multivariate analysis. The level of statistical significance was set at *p* ≤ 0.05. Statistical analyses were performed using Stata, version 15 (Stata Corp, College Station, TX). Qualitative analysis was done using content thematic analysis of the qualitative responses; coding the interview data to represent themes or new emerging themes.

## Results

In total 402 Mukuru residents participated in the study (Table 1). The majority were female (71.9%, *n* = 289) and younger than 40 years of age (79.6%, *n* = 320). More than half had at least secondary education (58.2% *n* = 234) or higher.

**Table 1:** Characteristics of participants and association with knowledge of climate change and perception of impact of climate changes on the community

Characteristics of participants		Heard of Climate Change before*		OR (95% CI)	***p***-value	Adjusted OR	***p***-value	Climate change affecting community#		OR (95% CI)	***p***-value	Adjusted OR	***p***-value
	*N* = 402	Yes (*n* = 307, 76.4%)	No (*n* = 95, 23.6%)					Yes (*n* = 365, 90.8%)	No (*n* = 37, 9.2%)				
Sex													
Female	*n* = 289 (71.9)	212 (73.4)	77 (26.6)	0.5 (0.3 – 0.9)	0.03	0.8 (0.4 – 1.6)	0.46	263 (91.0)	26 (9.0)	1.1 (0.5 – 2.2)	0.82		
Male	*n* = 114 (28.1)	95 (84.1)	18 (15.9)	Ref.	Ref.			102 (90.3)	11 (9.7)	Ref.	Ref.		
Age													
18-30 years	*n* = 208 (51.7)	154 (74.0)	54 (26.0)	Ref.	Ref.			184 (88.5)	24 (11.5)	Ref.	Ref.		
31-40 years	*n* = 112 (27.9)	87 (77.7)	25 (22.3)	1.2 (0.7 2.1)	0.47			104 (92.9)	8 (7.1)	1.7 (0.7 – 3.9)	0.22		
41-50 years	*n* = 52 (12.9)	42 (80.8)	10 (19.2)	1.5 (0.7 – 3.1)	0.32			48 (92.3)	4 (7.7)	1.6 (0.5 – 4.7)	0.43		
> 51 years	*n* = 30 (7.4)	24 (80.0.)	6 (20.0)	1.4 (0.5– 3.6)	0.48			29 (96.7)	1 (3.3)	3.8 (0.5 – 29.0)	0.20		
Highest level of education													
No formal education	*n* = 10 (2.5)	7 (70.0)	3 (30.0)	Ref.	Ref.			9 (90.0)	1 (10.0)	Ref.	Ref.		
Primary education	*n* = 144 (35.8)	99 (68.8)	45 (31.2)	0.9 (0.2 – 3.8)	0.93			130 (90.3)	14 (9.7)	1.0 (0.1 – 8.8)	0.98		
Secondary education	*n* = 203 (50.5)	162 (79.8)	41 (20.2)	1.7 (0.4 – 6.8)	0.46			187 (92.1)	16 (7.9)	1.3 (0.2 – 10.9)	0.81		
Undergraduate/bachelor’s degree or higher	*n* = 31 (7.7)	28 (90.3)	3 (9.7)	4 (0.7 – 24.2)	0.13			28 (90.3)	3 (9.7)	1.0 (0.1 – 11.3)	0.98		
Other	*n* = 14 (3.5)	11 (78.6)	3 (21.4)	1.6 (0.2 – 10.1)	0.63			11 (78.6)	3 (21.4)	0.4 (0.03 – 4.6)	0.47		
Current occupation													
Student	*n* = 14 (3.5)	12 (85.7)	2 (14.3)	1.4 (0.3 – 7.4)	0.67	1.9 (0.3 – 11.3)	0.47	12 (85.7)	2 (14.3)	0.1 (0.01 – 1.3)	0.08	0.2 (0.01 – 2.2)	0.18
Manager/supervisor/businessman/woman	*n* = 123 (30.6)	93 (75.6)	30 (2.4)	0.7 (0.3 – 1.6)	0.45	1.0 (0.4 – 2.3)	0.93	111 (90.2)	12 (9.8)	0.2 (0.02 – 1.3)	0.09	0.2 (0.02 – 1.7)	0.16
Housewife or domestic worker	*n* = 87 (21.6)	57 (65.5)	30 (34.5)	0.5 (0.2 – 1.0)	0.05*	0.7 (0.3 – 1.7)	0.40	77 (8.5)	10 (11.5)	0.1 (0.01 – 1.1)	0.06	0.2 (0.03 – 1.9)	0.14
Casual worker	*n* = 76 (18.9)	65 (85.5)	11 (1.5)	1.4 (0.6 – 3.5)	0.46	1.0 (0.4 – 2.3)	0.93	71 (93.4)	5 (6.6)	0.3 (0.03 – 2.2)	0.22	0.4 (0.04 – 3.5)	0.39
Unemployed	*n* = 57 (14.2)	46 (80.7)	11 (19.3)	Ref.	Ref.	Ref.	Ref.	56 (98.2)	1 (1.8)	Ref.	Ref.	Ref.	Ref.
Other (e.g., Industrial worker, Technician, Craft and related trade worker, Service and sales worker, Support staff, Agricultural worker)	*n* = 45 (11.2)	34 (75.6)	11 (24.4)	0.7 (0.3 – 1.9)	0.53	0.6 (0.2 – 1.9)	0.40			0.1 (0.01 – 0.8)	0.03*	0.1 (0.01 – 1.1)	0.06
Years lived in Mukuru													
0-5 years	*n* = 135 (33.6)	91 (67.4)	44 (32.6)	Ref.	Ref.	Ref.	Ref.	118 (87.4)	17 (12.6)	Ref.	Ref.	Ref.	Ref.
6-10 years	*n* = 81 (20.2)	61 (75.3)	20 (24.7)	1.5 (0.8 – 2.7)	0.22	1.7 (0.8 – 3.4)	0.17	71 (87.7)	10 (12.3)	1.0 (0.4 – 2.4)	0.96	0.9 (0.4 – 2.3)	0.85
> 10 years	*n* = 186 (46.3)	155 (83.3)	31 (16.7)	2.4 (1.4 – 4.1)	0.001*	3.1 (1.7 – 5.8)	< 0.001*	176 (94.6)	10 (5.4)	2.5 (1.1 – 5.7)	0.03*	2.6 (1.1 – 6.1)	0.03*
Number of people living in household													
One (respondent lives alone)	*n* = 50 (12.4)	43 (86.0)	7 (14.0)	Ref.	Ref.	Ref.	Ref.	45 (90.0)	5 (10.0	Ref.	Ref.		
Less than 5	*n* = 231 (57.5)	176 (76.2)	55 (23.8)	0.5 (0.2 – 1.2)	0.14	0.6 (0.2 – 1.5)	0.24	205 (88.7)	26 (11.3)	0.9 (0.3 – 2.4)	0.80		
5-10 persons	*n* = 119 (29.6)	86 (72.3)	33 (27.7)	0.4 (0.2 – 1.0)	0.06*	0.4 (0.1 – 1.0)	0.06	113 (95.0)	6 (5.0)	2.1 (0.6 – 7.2)	0.24		
> 10 persons	*n* = 2 (0.5)	2 (100.0)	0 (0.0)	–	–	–	–	2 (100.0)	0 (0.0)	–			
House/living situation													
Rent	*n* = 306 (89.6)	273 (75.8)	87 (24.2)	Ref.	Ref.			36 (90.0)	4 (10.0)	Ref.	Ref.		
Own	*n* = 40 (10)	32 (80.0)	8 (20.0)	0.8 (0.3 – 1.8)	0.56			327 (90.9)	33 (9.2)	1.1 (0.4 – 3.3)	0.86		
Sublet/lease	*n* = 2 (0.5)	2 (100.0)	0 (0.0)	–	–			2 (100.0)	0 (0.0)	–	–		
Location of house													
Flat area	*n* = 177 (44)	119 (67.2)	58 (32.8)	Ref.	Ref.	Ref.	Ref.	156 (88.1)	21 (11.9)	Ref.	Ref.	Ref.	Ref.
Low-lying area	*n* = 110 (27.4)	105 (95.4)	5 (4.6)	0.8 (0.4 – 1.4)	0.39	0.8 (0.4 – 1.5)	0.43	46 (78.0)	13 (22.0)	0.5 (0.2 – 1.0)	0.06	0.5 (0.2 – 1.1)	0.09
Riverside	*n* = 59 (14.7)	36 (61.0)	23 (39.0)	10.2 (4.0 – 26.5)	< 0.001*	10.7 (4.0 – 28.4)	< 0.001*	108 (98.2)	2 (1.8)	7.3 (1.7 – 31.6)	0.008*	7.7 (1.7 – 34.0)	0.007*
Steep incline/hill	*n* = 56 (13.9)	47 (83.9)	9 (16.1)	2.5 (1.2 – 5.5)	0.02*	2.1 (0.9 – 4.7)	0.08	55 (98.2)	1 (1.8)	7.4 (1.0 – 56.3)	0.05*	5.7 (0.7 – 44.0)	0.09
Roofing material													
Zinc/metal roofing	*n* = 375 (93.3)	283 (75.5)	92 (24.5)	Ref.	Ref.			340 (90.7)	35 (9.3)	Ref.	Ref.		
Concrete roofing	*n* = 11 (2.7)	10 (90.9)	1 (9.1)	3.3 (0.4 – 25.7)	0.26			10 (90.9)	1 (10.1)	1.0 (0.1 – 8.3)	0.98		
Makeshift	*n* = 9 (2.2)	9 (100.0)	0 (0.0)	–	–			9 (100.0)	0 (0.0)	–	–		
Other material	*n* = 7 (1.7)	5 (71.4)	2 (28.6)	0.8 (0.2 – 4.3)	0.81			6 (85.7)	1 (14.3)	0.6 (0.1 – 5.3)	0.66		
Wall material (Outer walls of house)													
Sheet metal	91,8 (*n* = 369)	277 (75.1)	92 (24.9)	Ref.	Ref.	Ref.	Ref.	333 (90.2)	36 (9.8)	Ref.	Ref.		
Concrete	*n* = 28 (7)	25 (89.3)	3 (10.7)	0.4 (0.1 – 1.2)	0.10	0.4 (0.1 – 1.5)	0.17	27 (96.4)	1 (3.6)	0.3 (0.04 – 2.6)	0.30		
Other material (Brick, Plywood, Other)	*n* = 5 (1.2)	5 (100.0)	0 (0.0)	–	–	–	–	5 (100.0)	0 (0.0)	–	–		
House insured against climate change hazards such as hurricane, flooding or other natural hazards													
No not insured	*n* = 290 (72.1)	219 (75.5)	71 (24.5)	Ref.	Ref.			262 (90.3)	28 (9.7)	Ref.	Ref.		
Yes insured	*n* = 7 (1.7)	5 (71.4)	2 (28.6)	1.2 (0.2 – 6.5)	0.80			7 (100.0)	0 (0.0)	–	–		
Don’t know/Not sure	*n* = 105 (26.1)	83 (79.0)	22 (21.0)	1.5 (0.3 – 8.3)	0.64			96 (91.4)	9 (8.6)	0.9 (0.4 – 1.9)	0.74		

*Ref* Reference

*variables statistically significant (*p* ≤ 0.05)

1) *Adjusted model for all variables *p* ≤ 0.1 (gender, current occupation, years lived in Mukuru, number of people in household, location of house, wall material)

2) #Adjusted model for all variables *p* ≤ 0.1 (current occupation, years lived in Mukuru, location of house)

3) ‘Adjusted model for all variables *p* ≤ 0.1 (years lived in Mukuru, location of house, wall materials, insured against climate hazards) – data for individual variables not shown in Table

Almost half of respondents had lived in Mukuru for more than 10 years (46.3% *n* = 186). Most respondents lived in a rented house (89.6% *n* = 306), with zinc/metal roofing (93.3% *n* = 375), sheet metal walls (91,8% *n* = 369) that was situated near to a riverside, low-lying area, or steep incline (56% *n* = 225).

Of respondents 76.4% (*n* = 307) had heard of climate change before the interview. Factors significantly associated with having heard of climate change before were having lived in Mukuru for more than 10 years (aOR 3.1; 95%CI 1.7 – 5.8; *p* < 0.001) and living in a dwelling close to the riverside (aOR 10.7; 95%CI 4.0 – 28.4, *p* < 0.001). The majority, 90.8% (*n* = 365), reported that climate change was affecting their community. Factors significantly associated with experiencing that climate change was affecting the community were, also, having lived in Mukuru for more than 10 years (aOR 2.6; 95%CI 1.1 – 6.1; *p* = 0.03) and living in a dwelling close to the riverside (aOR 7.7; 95%CI 1.7 – 34.0; *p* = 0.007). 82.6% (*n* = 332) of respondents were concerned about climate change, while 92.6% (*n* = 372) were concerned with the health-related impact of climate change. Those having lived in Mukuru for more than 10 years (vs. ≤6 years; aOR’ 2.5; 95%CI 1.1 – 5.8; *p* = 0.04) as well as those living at the riverside (vs. flat areas; aOR’ 7.7; 95%CI 1.7 – 34.2; *p* = 0.007) were significantly more likely to be concerned with the health-related impacts of climate change.

**Fig. 1:**
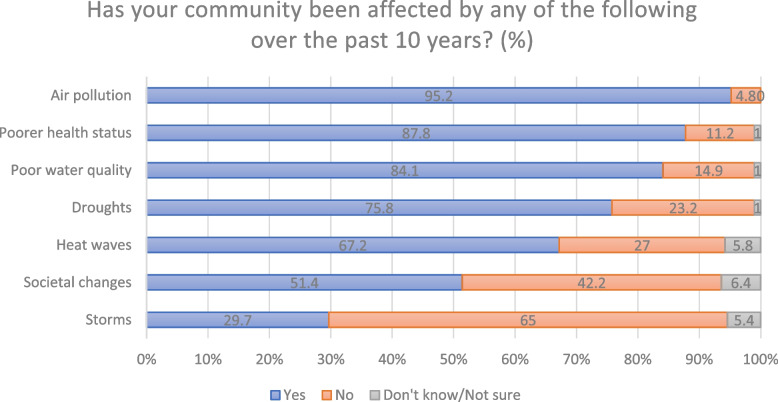
Percent of respondents stating that their community, within the last 10 years, has been affected by …

Figure 1 show the proportions of respondents indicating that their community, has within the last 10 years, been affected by different climate change related impacts. Asked what the respondents considered the most important climate change related issues among those experienced (Fig. 1) affecting the community on a scale from 1 to 10 (from 1 most important to 10 least important – results here reported as Median; Inter-Quartile Range (IQR)) they answered air pollution (1; IQR 1-3); poorer health status (e.g. undernutrition, respiratory diseases, cardiovascular disease, infectious diseases, poisoning) (2; IQR 1-4); droughts (3; IQR 1-5); heat waves (4; IQR 2-8); societal; changes (e.g. need to move, increased conflict status) (6; IQR 3-10); ecological changes (e.g. loss of animals and plants) (7; IQR 4-10); changes in use of land (e.g. differences in seasonality of crops) (8; IQR 5-10); and storms (9 IQR 5-9). When asked how climate change is affecting their community, most respondents mentioned diseases (infectious air- and water-borne diseases like flu, cold, typhoid, cholera, malaria as well as respiratory diseases like coughing, pneumonia and asthma) and air and water pollution from surrounding factories and lack of sanitation and drainage. In addition, insecure food supplies, rising food prices and water shortage was by many connected to extreme weather events like heat waves, floods, and droughts. Floods were mentioned to cause a rise in water-borne illnesses, malaria, and skin rashes due to unclean water. Also insecure housing situations vulnerable to floods not insulated creating an unbearably hot indoor environment in the metal sheet houses during the hot season and very cold during the cold were mentioned:

“*Houses being swept away due to heavy rains.*”

“*Excessive heat during hot seasons.*”

“*When it is sunny it is very uncomfortable to stay in our houses that are made of metal sheet.*”

“*Houses are made of metal sheet that doesn’t keep the heat.*”

**Fig. 2:**
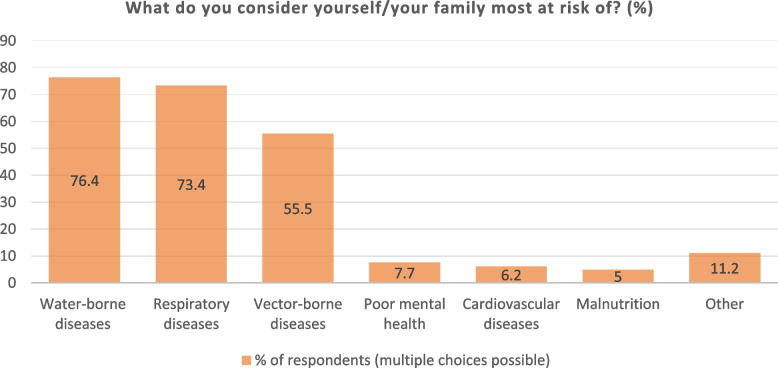
Perceived health risks among Mukuru residents (in general)

Perceived general health risks (not only climate change-related) among Mukuru residents are shown in Fig. 2. Most consider their families at risk of water-borne diseases (76.4% *n* = 307), respiratory diseases (73.4% *n* = 295) and vector-borne diseases (55.5% *n* = 223), followed by poor mental health (7.7% *n* = 31), cardiovascular diseases (6.2% *n* = 25) and malnutrition (5% *n* = 20). Other risks mentioned were headaches, tuberculosis, polio, HIV and other STDs, as well as disabilities and physical injuries. Asked what illnesses climate change in specific increases the risk of getting (multiple choices possible), respondents answered respiratory diseases e.g. cough a lung cancer (82.1% *n* = 330), infectious diseases e.g. diarrhoea, cholera, typhoid (74,1% *n* = 298), vector-borne diseases e.g. malaria, bilharzia (56,5% *n* = 227), malnutrition e.g. undernutrition (7,5% *n* = 30), and cardiovascular diseases e.g. high blood pressure, stroke, heart attack (7,2% *n* = 29).

**Fig. 3:**
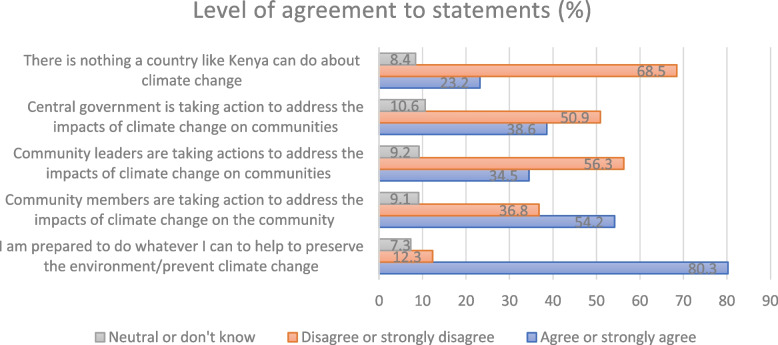
Perceived actors to address climate change and related health risks according to Mukuru residents

Figure 3 shows that the majority (68.5% (*n* = 269)) do believe that a country like Kenya can do something about climate change. Most respondents disagree that community leaders and the central government are taking action to address the impacts of climate change on the community. Asked if the central government in Kenya is taking action to address the impacts of climate change on communities 50.9% (*n* = 203) either disagree or strongly disagree; 10.6% (*n* = 42) were neutral or not sure; and 38.6% (*n* = 154) either agree or strongly agree. However, a large majority of respondents (80.3% (*n* = 318)) are prepared to do whatever they can to help preserve the environment/prevent climate change.

Asked if the community has taken action to prevent or lessen the health impact of climate change in their community, 44% (*n* = 177) say “*Yes*”, while 24.6% (*n* = 99) say “*Do not have enough information about climate change and health-related issues*”, 23.1% (*n* = 93) say “*Not aware of what actions can/should be taken*”, 3.7% (*n* = 15) say “*Climate change related health issues is not a concern in the community*”; and only 2.5% (*n* = 10) say “*It is not our responsibility to take action*”.

Of the respondents 65.9% (*n* = 265) stated that the government is the main agent responsible for addressing climate change in Kenya. Further responses to that question were: Everyone 32.1% (*n* = 129); Community organizations 28.1% (*n* = 113); Private citizens 15.4% (*n* = 62); Developed countries/United Nations (UN)/Industry 3.9% (*n* = 16); or Other/Don’t know 17.2% (*n* = 68).

Many respondents said that more information on climate change is generally needed among the residents in Mukuru. Of the respondents 96.8% (*n* = 388) would like to learn more about climate change, and 98.5% (*n* = 391) would like to learn about climate change related health impacts. These results indicate that residents in the informal settlement Mukuru are willing to act and change practices to adapt to and mitigate impacts of climate changes. This suggests that residents in slums believe that their local actions matter in the prevention and mitigation on climate change impacts.

Given the chance to add something at the end of the interview many respondents (*n* = 43) directly requested climate change education, training, or sensitization in their community:

“*When are you coming to educate us on climate change?*”

“*We need to be trained on (what) actions to take.*”

Asked what was needed to support climate adaptation and mitigation efforts in Mukuru more information, education and training on climate change, its health impacts, and ways to protect themselves and the community were requested by the respondents in our study. This suggests that advocacy, training or capacity building for improved housing and slum upgrading is needed now as the residents are concerned, experience climate change adverse health effects and are thus eager to get empowered to act.

Further, centrally organised community clean ups of the excessive amount of trash were requested to improve the water infrastructure. Currently children living in Mukuru play in the unclean, clogged drainages. When it rains the water floods into the houses, and the tanks containing drinking water gets contaminated, and thus government action was requested:

“*The government should provide clean water to the people of my community and ways to dispose garbage.*”

The government and local leaders were by many respondents (*n* = 110) asked to take responsibility and action to mitigate the effects of climate change and related health risks by unblocking drainage systems during floods to reduce water-borne diseases and flooding of housing, as well as providing safe drinking water both during floods and droughts. A further issue raised was the urbanization causing Mukuru to grow in population size making the housing and health conditions and climate change vulnerability grow. Why informal settlements like Mukuru in Nairobi, Kenya keep growing is exemplified by one respondent who told why he/she moved to Mukuru:

“*I relocated to Nairobi because of climate change affected me due to lack of rains, I stopped farming and animal farming to come (to Nairobi) and look for employment.”*

## Discussion

A main finding of this study is that the residents in the informal settlement Mukuru, Nairobi, Kenya are aware of climate change and its effects, experience climate change affects their community, and are concerned about both climate change and climate change related health impacts [7, 11, 14, 16, 18, 27]. This high degree of knowledge is consistent with recent studies e.g. in South African informal settlements [28] and a qualitative study conducted in Mukuru [18] where health care workers, community leaders and volunteers living in Mukuru were all conscious of a link between climate change and health [18] which correlates with the findings of our study.

In this study all participants consider themselves and their families at risk of respiratory, water- and vector-borne diseases; all diseases which have also been associated with climate change induced weather in previous studies [7, 11, 14, 16, 18, 27, 29, 30]. Other studies and reports have stated that over the last 10 years air pollution, a decrease in general health, poorer water quality, droughts and heat waves were the most experienced impacts of climate change on Mukuru informal settlements [18, 20, 31].

Interestingly, having lived in Mukuru for more than 10 years, and living close to the riverside was significantly associated with having heard of climate change before our study, experiencing impact of climate changes on the community and being concerned with health-related impacts of climate change when controlling for confounders. This could be explained by the fact that having lived long in Mukuru, the chances rise of having experienced the change of climate over time (increase in extreme weather events such as floods, heat waves, drought, increasing air and water pollution) and living close to a river side having your house being more directly affected by floods which makes you more vulnerable and thus aware of climate change.

Slum upgrading is important to fight health risk factors in Mukuru [20, 31]. All the climate change related health risks that are considered main risks (Fig. 1) might be mitigated through slum upgrading [11, 12, 32]. This is in line with findings in a study where Tanzanian informal settlement residents were found heat-health vulnerable [29, 33]. Housing improvements of the metal sheet houses would increase health as better housing can prevent heat shock (summer), cold (winter) and more stable housing during rainy seasons could also reduce stress and insecurities [34]. Other studies similarly find that upgrading of infrastructure including roads, drainage systems, sanitary facilities and dump sites will further make the vulnerable people living in informal settlements less vulnerable to extreme climate change related weather events [8, 29, 33, 35, 36].

Raising communal voices on climate change challenges at the community level on short-term projects like garbage collection or cleaning the drainage systems could be easily implemented. Community capacity building may be done through educating and sensitizing local groups to be knowledgeable ambassadors disseminating information on climate change and related health risks and mobilising local mitigation strategies [28, 37]. Training e.g., active youth or women groups, Community Health Volunteers (CHVs) or Community Health Workers (CHWs) may be a strategy [20]. In other low resource settings, CHV/CHW are used to help improve health outcomes in many different areas [38–40]. Further research is needed as to how adapting a similar strategy to the field of climate change and related health impacts may be sustainable in informal settlements like Mukuru, Nairobi in the future.

### Strengths and limitations

The findings of the study are limited to the specified areas, groups, and setting. The interview settings inside or in front of the private homes of each respondent, ensured that they could speak and respond freely with no or little interference. Before data collection the principal investigator, with support from the NCDAK, trained and instructed the research assistants. All research assistants had experience with data collection and good interviewing and interpersonal skills that are crucial to establish trust with the participant.

Since 71.9% (*n* = 289) of respondents were women, possible selection bias in recruiting must be taken into consideration. The research assistants performing the data collection reported this gender distribution to be caused by mostly women being at home with the kids during the daytime when the data collection was performed. Serious efforts were made to recruit as many men as possible in the study, but some men were asleep after working overnight shift in the surrounding factories; some men were not willing to prioritize the time for the interview; and in Mukuru men often sit in groups (some drinking alcohol) and many were not willing to leave the group to go somewhere quiet for the interview. This possible selection bias could lead to an overestimation of climate change knowledge if women know more about climate change effects. But to the best of our understanding, men and women alike in Mukuru have the same information level about the current local situation and challenges.

During the weeks of data collection (September 2021) there was a lot of tension in Mukuru due to upcoming elections, unrest caused by dissatisfaction with ongoing COVID-19 restrictions, as well as due to recent government demolition of hundreds of household structures to make room for a new highway across the area. This might have caused some suspicion about the reason and sincerity of the study among possible participants, which could lead to selection bias. However, this was addressed by close communication with the local chief officers about the study aim and conducting of the data collection.

Security during data collection was a possible challenge, as in some places of Mukuru a risk of getting mugged exist especially if carrying a (data entry) tablet. The research assistants felt responsible for the tablets handed to them, so they might have refrained from entering the most unsafe parts of Mukuru. This could have led to an overestimation of the climate change knowledge if knowledge is less in the less safe part of Mukuru. We strove to mitigate this risk of bias through the data collection method described (spinning a pen, Kish method), and we kept the survey teams safe by having a local Mukuru guide accompany them at all times.

A possible information bias may have arisen due to the language barriers. Though the questionnaire was in English, most of the interviews had to be conducted in the local Swahili or Sheng. Since there is no direct translation of “climate change” to Swahili or Sheng, the research assistants reported it difficult to explain the research topic and especially the elderly respondents had difficulties describing climate change in Swahili in their own words. This may have led to an underestimation of reported climate change, but as the local guides were able to assist in the translation, and as we find a high degree of knowledge and awareness about climate change and its health-related impacts, we regard this bias relatively low.

## Conclusions

The residents in Mukuru informal settlement, Nairobi, Kenya have high knowledge about climate change and its health-related impacts. Their attitude towards and motivation to learn about climate change and related health risks to be able to mitigate the effects is present and high. Having lived in the informal settlement more than 10 years and living close to the riverside was significantly associated with having knowledge of climate change, experiencing impact of climate changes on the community, and being concerned with health-related impacts of climate change when controlling for confounders.

The findings from this study may be used to identify ways in which knowledge on climate change resilience, in particular related to health, can be improved and how education can be tailored to the local context. Further, this study provides insights which may prove useful for policy makers, intervention planners and researchers as increased recognition of the impact of climate change on health advocates for local level climate change mitigation and adaption strategies. Thus, the findings may help inform appropriate interventions on climate change mitigation and adaptation as well as local, regional, and national policy making.

## Data Availability

The dataset generated and/or analysed during the current study are not publicly available to protect the participants as data may be tracked back to suburbs which can have political implications. Data that support the findings of this study are however available from the corresponding author upon reasonable request.

## Abbreviations

CD: Communicable Diseases
CHV: Community Health Volunteer
CHW: Community Health Worker
CISU: Civil Society in Development
DANIDA: Danish Ministry of Foreign Affairs; Danish International Development Agency
DNCDA: Danish Non-Communicable Diseases Alliance
ESRC: Amref Ethics and Scientific Review Committee
FGD: Focus Group Discussion
KAP: Knowledge, Attitudes and Practices
LMICs: Low- and Middle-Income Countries
NCDs: Non-Communicable Diseases
NCDAK: Non-Communicable Diseases Alliance of Kenya
NGO: Non-Governmental Organization
SDG: Sustainable Development Goal
SSA: Sub-Sahara Africa
UN: United Nations
WHO: World Health Organization
